# 3-[(Cyclo­hexyl­idene)amino]-1-(4-methyl­phen­yl)thio­urea

**DOI:** 10.1107/S1600536811015510

**Published:** 2011-04-29

**Authors:** Yan-Ling Zhang, Xiao-Wei Zhang, Fu-Juan Zhang

**Affiliations:** a College of Chemistry and Chemical Engineering, Xuchang University, Xuchang, Henan Province 461000, People’s Republic of China

## Abstract

In the title compound, C_14_H_19_N_3_S, the cyclo­hexane ring has a chair conformation. The almost planar amino­thio­urea unit (r.m.s. deviation = 0.0062 Å) is aligned at a dihedral angle of 45.23 (8)° with respect to the benzene ring. Inter­molecular N—H⋯N and N—H⋯S hydrogen bonding stabilizes the crystal structure.

## Related literature

For related structures and the biological applications of thio­semicarbazones, see: Hu *et al.* (2006 ▶).
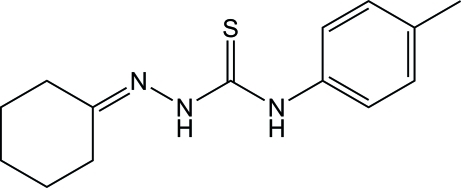


## Experimental

### 

#### Crystal data

C_14_H_19_N_3_S
*M* *_r_* = 261.38
Orthorhombic, 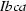

*a* = 14.9151 (4) Å
*b* = 22.5593 (5) Å
*c* = 17.1642 (3) Å
*V* = 5775.3 (2) Å^3^
*Z* = 16
Cu *K*α radiation
μ = 1.87 mm^−1^
*T* = 291 K
0.40 × 0.25 × 0.25 mm

#### Data collection

Oxford Diffraction Xcalibur Eos Gemini diffractometer
Absorption correction: multi-scan (*CrysAlis RED*; Oxford Diffraction, 2010 ▶) *T*_min_ = 0.521, *T*_max_ = 0.652
7202 measured reflections
2583 independent reflections
2024 reflections with *I* > 2σ(*I*)
*R*_int_ = 0.032

#### Refinement

*R*[*F* ^2^ > 2σ(*F* ^2^)] = 0.053
*wR*(*F* ^2^) = 0.162
*S* = 1.02
2583 reflections
172 parameters
H atoms treated by a mixture of independent and constrained refinement
Δρ_max_ = 0.37 e Å^−3^
Δρ_min_ = −0.25 e Å^−3^

### 

Data collection: *CrysAlis PRO* (Oxford Diffraction, 2010 ▶); cell refinement: *CrysAlis PRO*; data reduction: *CrysAlis RED* (Oxford Diffraction, 2010 ▶); program(s) used to solve structure: *SHELXTL* (Sheldrick, 2008 ▶); program(s) used to refine structure: *SHELXTL*; molecular graphics: *SHELXTL*; software used to prepare material for publication: *SHELXTL*.

## Supplementary Material

Crystal structure: contains datablocks I, global. DOI: 10.1107/S1600536811015510/xu5197sup1.cif

Structure factors: contains datablocks I. DOI: 10.1107/S1600536811015510/xu5197Isup2.hkl

Supplementary material file. DOI: 10.1107/S1600536811015510/xu5197Isup3.cml

Additional supplementary materials:  crystallographic information; 3D view; checkCIF report

## Figures and Tables

**Table 1:** Hydrogen-bond geometry (Å, °)

*D*—H⋯*A*	*D*—H	H⋯*A*	*D*⋯*A*	*D*—H⋯*A*
N1—H1⋯N3^i^	0.89 (3)	2.48 (3)	3.268 (3)	148 (2)
N2—H2⋯S1^ii^	0.86 (3)	2.70 (3)	3.531 (2)	164 (3)

Symmetry codes: (i) 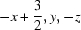; (ii) 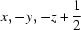.

**Fig. 1.**
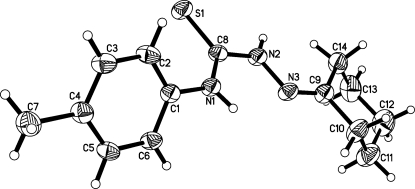
The molecular structure of the title compound, with displacement ellipsoids drawn at 30% probability level.

## supplementary crystallographic information

### Comment

Thiosemicarbazones have attracted much attention as they show potential application in the biological field (Hu *et al.*, 2006). There are a few single-crystal reports about them. Detailed information on their molecular and crystal structures is necessary to understand their anticancer activity. The molecular structure of (I) is shown in Fig 1. The cyclohexane ring adopts a chair conformation. The almost planar aminothiourea unit (r.m.s. deviation = 0.0062 Å) is aligned at a dihedral angle of 45.23 (8)° with respect to the plane of the benzene ring. In the crystal structure of the title compound, there are N—H···N an N—H···S hydrogen-bond interactions (Table 1).

### Experimental

*N*-(*p*-Tolyl)thiosemicarbazide (1.8 g, 10 mmol) and cyclohexanone (0.98 g, 10 mmol) was dissolved in 95% ethanol (15 ml) and the solution was refluxed for 0.5 h. Fine colorless crystals appeared on cooling. They were filtered and washed by 95% ethanol to give 1.6 g of the title compound in 61.5% yield. Single crystals suitable for X-ray measurements were obtained from methanol by slow evaporation at room temperature.

### Refinement

Imino H atoms were located in a difference Fourier map and refined isotropically. Other H atoms were placed in calculated positions with C—H = 0.93–0.97 and refined using a riding model, *U*_iso_(H) = 1.5*U*_eq_(C) for methyl H atoms and 1.2*U*_eq_(C) for the others.

### Crystal data

**Table:**

C_14_H_19_N_3_S	*F*(000) = 2240
*M**_r_* = 261.38	*D*_x_ = 1.202 Mg m^−^^3^
Orthorhombic, *I**b**c**a*	Cu *K*α radiation, λ = 1.54184 Å
Hall symbol: -I 2b 2c	Cell parameters from 2807 reflections
*a* = 14.9151 (4) Å	θ = 3.2–70.3°
*b* = 22.5593 (5) Å	µ = 1.87 mm^−^^1^
*c* = 17.1642 (3) Å	*T* = 291 K
*V* = 5775.3 (2) Å^3^	Prismatic, colorless
*Z* = 16	0.40 × 0.25 × 0.25 mm

### Data collection

**Table:**

Oxford Diffraction Xcalibur Eos Gemini diffractometer	2583 independent reflections
Radiation source: fine-focus sealed tube	2024 reflections with *I* > 2σ(*I*)
graphite	*R*_int_ = 0.032
ω scans	θ_max_ = 67.1°, θ_min_ = 3.9°
Absorption correction: multi-scan (*CrysAlis RED*; Oxford Diffraction, 2010)	*h* = −7→17
*T*_min_ = 0.521, *T*_max_ = 0.652	*k* = −26→26
7202 measured reflections	*l* = −19→20

### Refinement

**Table:**

Refinement on *F*^2^	Primary atom site location: structure-invariant direct methods
Least-squares matrix: full	Secondary atom site location: difference Fourier map
*R*[*F*^2^ > 2σ(*F*^2^)] = 0.053	Hydrogen site location: inferred from neighbouring sites
*wR*(*F*^2^) = 0.162	H atoms treated by a mixture of independent and constrained refinement
*S* = 1.02	*w* = 1/[σ^2^(*F*_o_^2^) + (0.1042*P*)^2^ + 0.7804*P*] where *P* = (*F*_o_^2^ + 2*F*_c_^2^)/3
2583 reflections	(Δ/σ)_max_ < 0.001
172 parameters	Δρ_max_ = 0.37 e Å^−^^3^
0 restraints	Δρ_min_ = −0.25 e Å^−^^3^

### Special details

**Table:**

Geometry. All e.s.d.'s (except the e.s.d. in the dihedral angle between two l.s. planes) are estimated using the full covariance matrix. The cell e.s.d.'s are taken into account individually in the estimation of e.s.d.'s in distances, angles and torsion angles; correlations between e.s.d.'s in cell parameters are only used when they are defined by crystal symmetry. An approximate (isotropic) treatment of cell e.s.d.'s is used for estimating e.s.d.'s involving l.s. planes.
Refinement. Refinement of *F*^2^ against ALL reflections. The weighted *R*-factor *wR* and goodness of fit *S* are based on *F*^2^, conventional *R*-factors *R* are based on *F*, with *F* set to zero for negative *F*^2^. The threshold expression of *F*^2^ > σ(*F*^2^) is used only for calculating *R*-factors(gt) *etc*. and is not relevant to the choice of reflections for refinement. *R*-factors based on *F*^2^ are statistically about twice as large as those based on *F*, and *R*- factors based on ALL data will be even larger.

### Fractional atomic coordinates and isotropic or equivalent isotropic displacement parameters (Å^2^)

**Table:**

	*x*	*y*	*z*	*U*_iso_*/*U*_eq_	
S1	0.88952 (5)	0.07068 (3)	0.17935 (3)	0.0599 (3)	
N1	0.84361 (13)	0.02381 (8)	0.04022 (10)	0.0481 (5)	
N2	0.78485 (14)	−0.01953 (8)	0.14881 (10)	0.0498 (5)	
N3	0.74084 (15)	−0.05790 (8)	0.09883 (10)	0.0514 (5)	
C1	0.89252 (14)	0.06343 (9)	−0.00784 (12)	0.0450 (5)	
C2	0.89188 (19)	0.12432 (11)	0.00239 (14)	0.0557 (6)	
H2A	0.8572	0.1413	0.0414	0.067*	
C3	0.94341 (19)	0.15961 (10)	−0.04609 (14)	0.0597 (6)	
H3	0.9435	0.2004	−0.0385	0.072*	
C4	0.99514 (19)	0.13567 (10)	−0.10592 (13)	0.0544 (6)	
C5	0.99072 (18)	0.07526 (10)	−0.11773 (13)	0.0520 (5)	
H5	1.0221	0.0585	−0.1590	0.062*	
C6	0.94050 (17)	0.03922 (10)	−0.06944 (12)	0.0505 (5)	
H6	0.9388	−0.0014	−0.0782	0.061*	
C7	1.0565 (2)	0.17332 (13)	−0.15541 (18)	0.0741 (8)	
H7A	1.0221	0.1928	−0.1951	0.111*	
H7B	1.1011	0.1486	−0.1793	0.111*	
H7C	1.0853	0.2025	−0.1233	0.111*	
C8	0.83781 (15)	0.02349 (9)	0.11831 (12)	0.0460 (5)	
C9	0.68926 (18)	−0.09724 (10)	0.12732 (14)	0.0538 (6)	
C10	0.6399 (2)	−0.13589 (14)	0.07092 (18)	0.0749 (8)	
H10A	0.6617	−0.1284	0.0186	0.090*	
H10B	0.5765	−0.1262	0.0723	0.090*	
C11	0.6526 (3)	−0.20137 (15)	0.0908 (2)	0.0911 (11)	
H11A	0.6166	−0.2254	0.0559	0.109*	
H11B	0.7149	−0.2122	0.0835	0.109*	
C12	0.6250 (3)	−0.21354 (16)	0.1747 (2)	0.0961 (11)	
H12A	0.5615	−0.2058	0.1808	0.115*	
H12B	0.6358	−0.2549	0.1869	0.115*	
C13	0.6773 (2)	−0.17499 (16)	0.2301 (2)	0.0873 (10)	
H13A	0.7401	−0.1861	0.2279	0.105*	
H13B	0.6563	−0.1820	0.2827	0.105*	
C14	0.6686 (2)	−0.10938 (14)	0.21164 (16)	0.0694 (7)	
H14A	0.6081	−0.0963	0.2232	0.083*	
H14B	0.7096	−0.0870	0.2443	0.083*	
H1	0.8188 (16)	−0.0081 (12)	0.0192 (16)	0.052 (7)*	
H2	0.799 (2)	−0.0305 (14)	0.195 (2)	0.075 (9)*	

### Atomic displacement parameters (Å^2^)

**Table:**

	*U*^11^	*U*^22^	*U*^33^	*U*^12^	*U*^13^	*U*^23^
S1	0.0824 (5)	0.0632 (4)	0.0341 (3)	−0.0172 (3)	−0.0022 (3)	−0.0052 (2)
N1	0.0605 (11)	0.0528 (9)	0.0309 (8)	−0.0072 (9)	−0.0017 (8)	−0.0002 (7)
N2	0.0634 (11)	0.0556 (10)	0.0305 (8)	−0.0083 (9)	0.0003 (9)	0.0003 (7)
N3	0.0674 (12)	0.0519 (9)	0.0349 (9)	−0.0070 (9)	−0.0031 (9)	−0.0016 (7)
C1	0.0521 (12)	0.0514 (11)	0.0316 (10)	−0.0003 (9)	−0.0032 (9)	0.0030 (8)
C2	0.0735 (15)	0.0532 (11)	0.0403 (11)	0.0110 (11)	0.0104 (11)	0.0009 (9)
C3	0.0864 (17)	0.0448 (11)	0.0478 (12)	0.0051 (11)	0.0066 (13)	0.0030 (9)
C4	0.0675 (14)	0.0561 (12)	0.0395 (11)	0.0009 (11)	0.0037 (11)	0.0078 (9)
C5	0.0638 (13)	0.0580 (12)	0.0344 (10)	0.0066 (11)	0.0062 (10)	0.0002 (9)
C6	0.0673 (14)	0.0482 (10)	0.0358 (10)	0.0004 (10)	−0.0008 (10)	−0.0013 (8)
C7	0.094 (2)	0.0640 (14)	0.0641 (16)	−0.0064 (15)	0.0204 (16)	0.0083 (13)
C8	0.0529 (12)	0.0526 (11)	0.0323 (10)	0.0015 (9)	0.0001 (9)	−0.0013 (8)
C9	0.0626 (14)	0.0548 (11)	0.0441 (12)	−0.0055 (11)	−0.0049 (11)	0.0044 (9)
C10	0.092 (2)	0.0761 (16)	0.0568 (15)	−0.0252 (16)	−0.0137 (15)	0.0025 (13)
C11	0.114 (3)	0.0713 (17)	0.088 (2)	−0.0287 (19)	0.002 (2)	−0.0052 (16)
C12	0.109 (3)	0.0764 (19)	0.103 (3)	−0.0260 (19)	0.007 (2)	0.0240 (19)
C13	0.088 (2)	0.102 (2)	0.0714 (19)	−0.0172 (19)	0.0029 (17)	0.0367 (18)
C14	0.0722 (16)	0.0869 (18)	0.0490 (14)	−0.0193 (15)	0.0081 (13)	0.0049 (13)

### Geometric parameters (Å, °)

**Table:**

S1—C8	1.681 (2)	C7—H7A	0.9600
N1—C8	1.343 (3)	C7—H7B	0.9600
N1—C1	1.418 (3)	C7—H7C	0.9600
N1—H1	0.89 (3)	C9—C10	1.497 (4)
N2—C8	1.356 (3)	C9—C14	1.505 (3)
N2—N3	1.384 (3)	C10—C11	1.528 (5)
N2—H2	0.86 (3)	C10—H10A	0.9700
N3—C9	1.272 (3)	C10—H10B	0.9700
C1—C2	1.385 (3)	C11—C12	1.523 (5)
C1—C6	1.389 (3)	C11—H11A	0.9700
C2—C3	1.385 (4)	C11—H11B	0.9700
C2—H2A	0.9300	C12—C13	1.506 (5)
C3—C4	1.393 (4)	C12—H12A	0.9700
C3—H3	0.9300	C12—H12B	0.9700
C4—C5	1.379 (3)	C13—C14	1.519 (5)
C4—C7	1.510 (4)	C13—H13A	0.9700
C5—C6	1.382 (3)	C13—H13B	0.9700
C5—H5	0.9300	C14—H14A	0.9700
C6—H6	0.9300	C14—H14B	0.9700
			
C8—N1—C1	128.10 (19)	N3—C9—C10	117.1 (2)
C8—N1—H1	112.0 (17)	N3—C9—C14	128.3 (2)
C1—N1—H1	119.4 (17)	C10—C9—C14	114.6 (2)
C8—N2—N3	118.98 (17)	C9—C10—C11	111.0 (3)
C8—N2—H2	115 (2)	C9—C10—H10A	109.4
N3—N2—H2	121 (2)	C11—C10—H10A	109.4
C9—N3—N2	119.01 (19)	C9—C10—H10B	109.4
C2—C1—C6	119.4 (2)	C11—C10—H10B	109.4
C2—C1—N1	123.2 (2)	H10A—C10—H10B	108.0
C6—C1—N1	117.39 (19)	C12—C11—C10	110.6 (3)
C3—C2—C1	119.4 (2)	C12—C11—H11A	109.5
C3—C2—H2A	120.3	C10—C11—H11A	109.5
C1—C2—H2A	120.3	C12—C11—H11B	109.5
C2—C3—C4	121.8 (2)	C10—C11—H11B	109.5
C2—C3—H3	119.1	H11A—C11—H11B	108.1
C4—C3—H3	119.1	C13—C12—C11	110.7 (3)
C5—C4—C3	117.7 (2)	C13—C12—H12A	109.5
C5—C4—C7	120.1 (2)	C11—C12—H12A	109.5
C3—C4—C7	122.1 (2)	C13—C12—H12B	109.5
C4—C5—C6	121.3 (2)	C11—C12—H12B	109.5
C4—C5—H5	119.4	H12A—C12—H12B	108.1
C6—C5—H5	119.4	C12—C13—C14	112.8 (3)
C5—C6—C1	120.3 (2)	C12—C13—H13A	109.0
C5—C6—H6	119.8	C14—C13—H13A	109.0
C1—C6—H6	119.8	C12—C13—H13B	109.0
C4—C7—H7A	109.5	C14—C13—H13B	109.0
C4—C7—H7B	109.5	H13A—C13—H13B	107.8
H7A—C7—H7B	109.5	C9—C14—C13	111.1 (3)
C4—C7—H7C	109.5	C9—C14—H14A	109.4
H7A—C7—H7C	109.5	C13—C14—H14A	109.4
H7B—C7—H7C	109.5	C9—C14—H14B	109.4
N1—C8—N2	115.25 (19)	C13—C14—H14B	109.4
N1—C8—S1	126.08 (17)	H14A—C14—H14B	108.0
N2—C8—S1	118.67 (16)		

### Hydrogen-bond geometry (Å, °)

**Table:**

*D*—H···*A*	*D*—H	H···*A*	*D*···*A*	*D*—H···*A*
N1—H1···N3^i^	0.89 (3)	2.48 (3)	3.268 (3)	148 (2)
N2—H2···S1^ii^	0.86 (3)	2.70 (3)	3.531 (2)	164 (3)

Symmetry codes: (i) −*x*+3/2, *y*, −*z*; (ii) *x*, −*y*, −*z*+1/2.
